# What Is the Use of Elephant Hair?

**DOI:** 10.1371/journal.pone.0047018

**Published:** 2012-10-10

**Authors:** Conor L. Myhrvold, Howard A. Stone, Elie Bou-Zeid

**Affiliations:** 1 Department of Civil and Environmental Engineering, Princeton University, Princeton, New Jersey, United States of America; 2 Department of Mechanical and Aerospace Engineering, Princeton University, Princeton, New Jersey, United States of America; University of Zurich, Switzerland

## Abstract

The idea that low surface densities of hairs could be a heat loss mechanism is understood in engineering and has been postulated in some thermal studies of animals. However, its biological implications, both for thermoregulation as well as for the evolution of epidermal structures, have not yet been noted. Since early epidermal structures are poorly preserved in the fossil record, we study modern elephants to infer not only the heat transfer effect of present-day sparse hair, but also its potential evolutionary origins. Here we use a combination of theoretical and empirical approaches, and a range of hair densities determined from photographs, to test whether sparse hairs increase convective heat loss from elephant skin, thus serving an intentional evolutionary purpose. Our conclusion is that elephants are covered with hair that significantly enhances their thermoregulation ability by over 5% under all scenarios considered, and by up to 23% at low wind speeds where their thermoregulation needs are greatest. The broader biological significance of this finding suggests that maintaining a low-density hair cover can be evolutionary purposeful and beneficial, which is consistent with the fact that elephants have the greatest need for heat loss of any modern terrestrial animal because of their high body-volume to skin-surface ratio. Elephant hair is the first documented example in nature where increasing heat transfer due to a low hair density covering may be a desirable effect, and therefore raises the possibility of such a covering for similarly sized animals in the past. This elephant example dispels the widely-held assumption that in modern endotherms body hair functions exclusively as an insulator and could therefore be a first step to resolving the prior paradox of why hair was able to evolve in a world much warmer than our own.

## Introduction

Elephants have a large heat transfer problem: they have the greatest volume-to- surface-area ratio of any terrestrial mammal [1], [2], [3] and they live in hot environments where temperatures can reach 50°C [4]. Prior studies have shown that even in a 30°C environment, an adult elephant needs to reject several kilowatts of heat averaged over a day [5], [6]. Known mechanisms of elephant heat transfer can be classified as behavioral such as ear flapping [7], dust bathing [8], moving to cooler shady areas [4], water-spraying, mud-spraying, and bathing [1], [9], or biophysical such as skin roughness [3], blood circulation through the ears [5], [10], [11], breathing and evaporation through the skin despite the lack of sweat glands [9], or body temperature fluctuations to store and release heat during different times of the day [12]. However, none of these mechanisms alone seem to be plausibly sufficient to fulfill an elephant’s heat transfer needs. Ear flapping and vasodilation are suggested major contributors to thermoregulation, but some estimates [11] put their contribution at only 8% of the standard metabolic rate (SMR). Furthermore, elephant ears are covered with hair, which means that any increase in the heat transfer rate due to hair will also affect the thermoregulatory role of the ears.

Under extreme heat conditions, such as when the air temperature exceeds the elephant’s body temperature (which fluctuates around 36°C [13]), it is clear elephants need behavioral mechanisms for thermoregulation (see Methods and Discussion S1). However, for a wide range of ordinary thermal conditions, biophysical factors can be sufficient to regulate the elephant’s body temperature. Heat loss by convection from the skin is one key biophysical thermoregulation mechanism [11]; nevertheless, it remains unclear if and how it can be affected by hair. The relative scarcity of elephant body hair on the skin surface has led many workers to erroneously assert that elephants are essentially hairless, when in fact overviews of elephant physiology note that hairs cover the entire body with varying hair lengths and densities [13]_,_ [14]. Postulated explanations have since suggested that hair is residual from evolution, where it may have served sensory or protective purposes (and may continue to do so today in the trunk and surrounding mouth area; a thermoregulatory role of elephant hair is not mutually exclusive with other functions).

**Figure 1:**
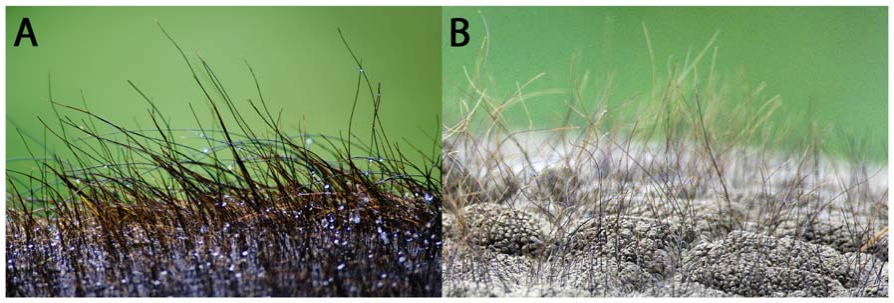
Pictures of elephant hair on the top of the back of an Asian elephant, (A) and an African elephant’s head (B). The presence of hair on elephants was first noted by van Leeuwenhoek [30]. Photos taken by Conor L. Myhrvold in the Woodland Park Zoo, Seattle, Washington, from outside of the elephant enclosure, with permission from the Zoo.

We provide examples of elephant hair in Fig. 1. Elephant hair diameters and lengths vary depending on type and location [13], and we use the representative values of 0.5 mm and 20 mm, respectively, for typical calculations (see Table 1 for a list of elephant hair and skin properties used here); but we also report results with different diameters in the Methods and Discussion S1. Qualitatively, Asian elephants (*Elephas maximus*) are notably hairier than their African counterparts (*Loxodonta africana, Loxodonta cyclotis*), and juvenile elephants have higher hair densities than adults [13]. Here we investigate the effect of body hair on the thermoregulation of the elephant and whether it is possible for hair to actually increase heat loss from the body. The rest of an elephant’s required heat loss is then performed through other means including radiative and latent losses (thermal energy used for water evaporation – such as breathing and evaporative cooling on the skin, but not sweating [9]).

**Table 1:** Elephant-Relevant Parameters.

Parameter	Value	Reference	Notes
Thermal conductivity – skin	0.475 W/(mK)	[31]	Skin frozen then thawed.
Thermal conductivity – hair	0.37–0.475 W/(mK); we use0.4 W/(mK)	[25]	Used same value of human skin for hair; range refers to what is encountered in the literature – see Methods and Discussion S1.
“Typical” hair diameter	0.5 mm	This study	See Methods and Discussion S1
“Typical” body hair length	20 mm	This study	See Methods and Discussion S1

## Materials and Methods

**Figure 2:**
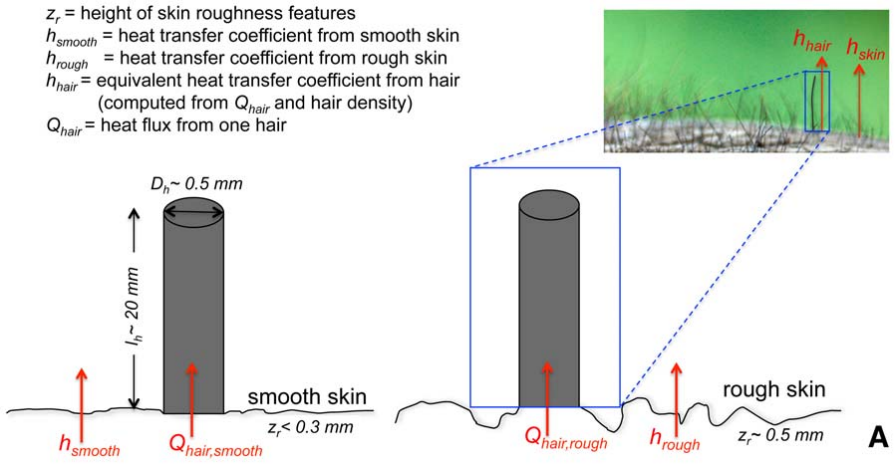
Schematic of our heat-transfer model showing model configuration and various parameters relating to rough and smooth skin (*individual hair in small box in top-right corner is enhanced for illustrative clarity*).

To quantify the effect of hair on thermoregulation, we compare the heat flux densities between the elephant skin with and without hair. We also account for the varying roughness of the skin between different elephants, and between different parts of the elephant body by studying a hydrodynamically smooth skin representative for example of the ears (in the air, this assumption typically corresponds to roughness features of less than 1 mm), as well as a hydrodynamically rough skin representative for example of the legs or back (roughness typically exceeding about 3 mm). Fig. 2 depicts a schematic of the heat fluxes and parameters we model, as detailed next.

Due to the low density of elephant hair, we assume that its presence does not significantly affect the air flow and temperature in the vicinity of the skin. Although in reality the presence of low-density hair will very slightly reduce the air speed and also slightly increase turbulence near the skin, these two effects are relatively small and have opposite impacts on heat exchange rates. Hairs can then be modeled as individual pin fins, of circular cross section, in an air flow bounded by the large body of the elephant (Fig. 2). A pin fin is a slender protrusion that increases the fluid-solid contact area and transfers heat into the surrounding moving fluid more efficiently since both the fluid velocity and the fluid-base surface temperature contrast increase away from the surface (see Methods and Discussion S1). Pin fins are widely studied in the engineering heat transfer literature.

The cases with and without hair are then studied as heat transfer problems from a flat plate of length 6 m and width 1 m since the elephant body has a large radius of curvature compared to the boundary layer thickness over the skin. This model represents, for example, one side of the elephant body and all calculations are normalized per unit area so that the exact size of the body is not relevant to our assessment. The heat fluxes from individual hairs in one unit area are then summed and compared to the heat flux from the skin without hair. Since we assume hairs do not change the flow over and heat exchange from the bare skin, the ratio of hair heat flux to bare skin heat flux is the fractional increase in heat transfer due to the presence of hair; this ratio is called “fin effectiveness” in the engineering literature.

For the smooth bare skin, we assume the air flowing over the flat skin surface already contains a range of turbulent eddies (air flow in the lower atmosphere is indeed highly turbulent) and we use the corresponding classic empirical relation [15], [16] for the Nusselt number (*Nu*), valid for the typical air velocities and elephant body sizes we encounter:
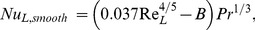
(1)where *Pr* is the Prandtl number (*Pr* = 0.714 = *ν*/*α*; *ν* = 1.603×10^−5^ m^2^/s is the kinematic viscosity and *α = *2.247×10^−5^ m^2^/s the thermal diffusivity of the air at 30°C). *Re_L_* = *U_air_ L/ν* is the (large) Reynolds number based on the elephant body length *L;* where *U_air_* is the wind speed away from the body, here referenced at 0.5 m; and *L* is taken as 6 m (length) but has little effect on the final results per unit area presented later. The parameter *B* is related to the distance (*x_cr_*) at which the boundary layer over the skin becomes a fully developed turbulent boundary layer. Using an expression of *B* as a function of a critical Reynolds number (based on *x_cr_*) provided in the literature [15], [16], we compute *B* to be about 180 taking into consideration that the inflow is turbulent and the development of a fully turbulent boundary layer is faster than with a laminar inflow (we thus use a critical Reynolds number of 1.1×10^5^). But we note that the results are not very sensitive to the exact value of *B*. *Nu_L_* given in Eq. 1 is the average Nusselt number based on *L* over the elephant body for smooth (and later for rough) skin, as indicated by the second subscript in Eq. 1, from which we can then obtain the heat transfer coefficient:
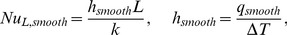
(2)Where *q_smooth_* is the heat flow rate per unit area from the bare smooth skin (i.e. heat flux density); *h_smooth_* is the heat transfer coefficient or the heat flux density per degree of temperature difference between the free stream air and the smooth skin; *k* is the thermal conductivity of the air (0.0263 W/(mK) at 30°C); and Δ*T* is the total temperature difference between the skin and the air. Thus a knowledge of *Nu_L,smooth_* allows us to determine *h_smooth_*, which is the basic parameter we will compare for cases with and without hair, and which does not depend on the temperature difference Δ*T*.

**Figure 3:**
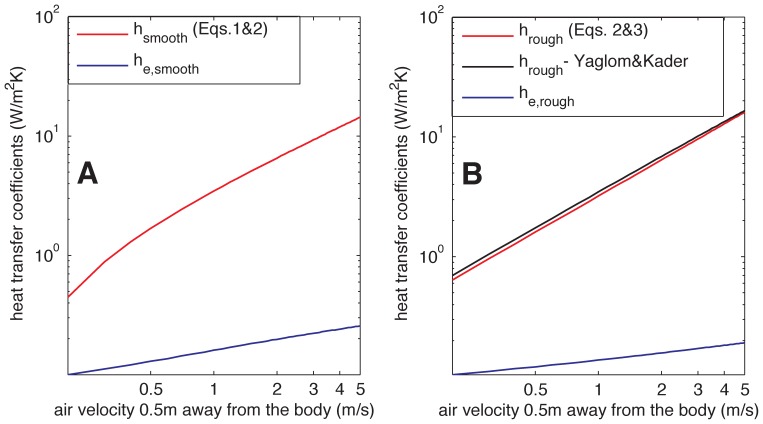
Variation of the different heat transfer coefficients with wind speed for a hair density of 1500 hairs/m^2^. (A) Heat transfer coefficient for bare smooth skin and effective heat transfer coefficient for hair over smooth skin. (B) Same as A, but for rough skin. B also depicts the alternative formulation for *h*_rough_ of Yaglom and Kader [18].

To obtain *h_rough_* for the bare rough skin, we can also use Eq. 2 but with the rough skin Nusselt number computed from a turbulent boundary layer similarity model:
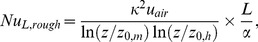
(3)where *κ* = 0.41 is the von-Karman constant; *u_air_* is the wind speed at some distance *z* from the surface, here we use *z* = 0.5 m; and *z_0,m_* and *z_0,h_* are the roughness lengths for momentum and heat of the surface, respectively. Typical values for these length scales are taken as *z_0,m_* = *z_r/_*10 and *z_0,h_* = *z_r/_*100 [17], where *z_r_* is the roughness of the elephant skin (≈5 mm for the rough parts). This model of *Nu* holds at a sufficiently large distance from the initial air-elephant contact point, but also provides a good estimate for smaller distances. It was also compared to a more elaborate model by Yaglom and Kader [18] for the rough-wall Nusselt number where the roughness lengths are not used explicitly and the results were very similar (Fig. 3). We thus mainly present and use the simpler formulation.

For the additional heat exchange with the atmosphere achieved by the hairs acting as fins, we use the solution for the 1D fin in a cross-flow, where the one-dimensional conduction along the fin is balanced by the lateral convection from the air flow around it. The total heat flux density from the base of a single fin (i.e. from the body of the elephant, see Fig. 2) is then given by [15]:
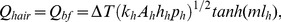
(4)where the *bf* subscript denotes the *b*ase of the *f*in; *A_h_, k_h_* ( = 0.4 W/(mK)), *p_h_*, and *l_h_* are the cross-sectional area, thermal conductivity, perimeter, and length of the hair, respectively (here we assume a hair diameter of 0.5 mm and a hair length of 20 mm); *m* = (*h_h_p_h_*/*k_h_A_h_*)^1/2^; and *h_h_* is the hair-fluid heat transfer coefficient. We note however that this model is not very sensitive to the hair length *l_h_*; mathematically *tanh*(*ml_h_*) asymptotes to 1 for large *l_h_* (the pin fin is called “long” when this factor is very close to 1). Physically, this represents the fact that the hair temperature decreases away from the skin and at some point it almost reaches the temperature of the air; the hair length beyond that point does not contribute to heat exchange. Our modeling results indicate that for elephant hair, the main part contributing to the heat exchange is the first 7 mm of hair closest to the skin, i.e. about one third of the 20 mm hair we model.

Since we are interested in the effective hair heat transfer coefficient per unit area of the elephant skin, we sum the fluxes from individual hairs over a unit area of elephant skin and normalize it by the temperature difference to define an effective heat transfer coefficient for hair (which is different for smooth and rough skin as detailed below) as illustrated in Fig. 2. This heat transfer coefficient is:
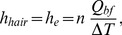
(5)where *n* is the hair density (hairs per m^2^). The only parameter that is not solely determined by the hair characteristic is the transfer coefficient *h_h_.* We compute that coefficient from well-established empirical relationships for a single cylindrical pin fin in a cross-flow, applicable over the whole range of Reynolds numbers (*Re_D_*) based on the hair diameter (*D_h_*) that our computations include. This Reynolds number is on the order of 100, which implies that the flow around and heat transfer from a single hair occur in the laminar regime, although the bulk flow on the scale of the elephant is turbulent. The heat transfer coefficient over the hair is [19]:
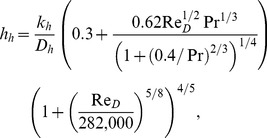
(6)where Dh is the hair diameter. This coefficient varies with the air speed around the hair and hence it varies along the hair since the wind speed varies. The variation of the wind speed with distance from the skin and with the ambient wind speed referenced at 0.5 m away from the body are calculated from well-established formulations for wall-bounded, steady-state flows [20]; more details are given in the Methods and Discussion S1.

After computing the variable *h_h_*(*z*) along the hair, an average value is used in Eq. 4; this simplification avoids the need to numerically solve the equation and did not have a large effect on the results in tests we performed where the average was taken over the whole hair or over the lowest one third of the hair. In the results that follow we use the average over the lowest third of the hair, which is the region contributing the most to heat exchange, since these results should be more accurate.

Hair density, *n* in Eq. 4., estimates are based on values we obtained from one reference individual (African elephant) using automated hair counting techniques_,_ and their range of variation is deduced by comparing pictures of different elephants to the picture of our reference individual, and accounting for the fact that Asian elephants have higher hair densities [13]. The automated hair counting approach was developed and applied to several pictures of a reference individual using Matlab™ routines (which can be obtained by contacting the authors). The approach involved identification of the hairs, filtering of outliers, and computation of the density statistics as detailed in the Methods and Discussion S1.

## Results

To illustrate the variations of the heat transfer coefficients with skin roughness and wind speed, and to evaluate the consistency and realism of our model, we plot all the computed heat transfer coefficients in Fig. 3 (note the log-log axes). In these figures, we observe that:

- the bare skin coefficient for the smooth surface is lower than for the rough skin and the effective hair coefficient over smooth skin is larger; the smooth skin is less efficient at exchanging heat with the fluid and also slows the fluid less than the rough skin, which implies that the hairs experience higher fluid velocities over a smooth skin and exchange more heat.
- As the wind speed *U* increases, the heat transfer rate from the rough skin, to a first order, increases ∼*U* (Eq. 3), the heat transfer rate from the smooth skin, to a first order, increases ∼*Re_L_* ^4/5^ ∼*U* ^4/5^ (Eq. 1), and the heat transfer rate from the hairs increase at most as *Re_D_* ^1/2^ ∼*U* ^1/2^ (Eq. 5, since the last term in that equation ≈ 1 for the range of *Re_D_* values we encounter in our computations; in fact the increase is even slightly lower due to the reduction in the length contributing to the heat transfer at higher wind speeds); this different scaling is responsible for the different slopes in Fig. 2, and suggests that with increasing *U* the relative effect of the hairs decreases (these results are confirmed in the next section).
- The rough skin coefficient (Eq. 3) and the alternative model we tested by Kader and Yaglom [18] yield very similar results.

Most importantly, these results can help to quantify the role of convective cooling in thermoregulation. A large adult elephant needs to dump energy at a rate of about 4500 W [5]. Assuming a 6 m long elephant with a diameter of the body of 3 m, with legs that are 2 m long and 0.4 m in diameter, and with 2×1 m ears, its skin surface would be about 88 m^2^. Thus the required heat flux density to release all the metabolic heat generation is 51 W/m^2^. At a wind speed of 3 m/s, the total heat exchange coefficient (bare skin+hair) we computed for a rough skin, for example, is ∼10 W/(m^2^K). Thus, for a 5°C difference between the free stream air and the elephant body temperature, convection alone can remove 100% of the metabolic heat production. However, under most conditions, radiation will also be an important cooling mechanism. With an air temperature of 12.6°C, Williams [11] estimated the contribution of convection to be about 40% of the total heat loss from the elephant, while radiative losses were estimated at about 50%.

To quantify the effect of hair on heat transfer and thermoregulation in elephants based on the developed models, the effective heat transfer coefficient *h_e_* (Eq. 4)_,_ which again is the additional heat transfer from the elephant body due to the hair only, is compared to the baseline (no-hair) coefficient for smooth (Eqs. 1 and 2)_,_ and rough (Eqs. 3 and 2) bare skins. The comparison of these heat transfer coefficients is easier to interpret since these coefficients normalize for the area and the temperature differences.

**Figure 4:**
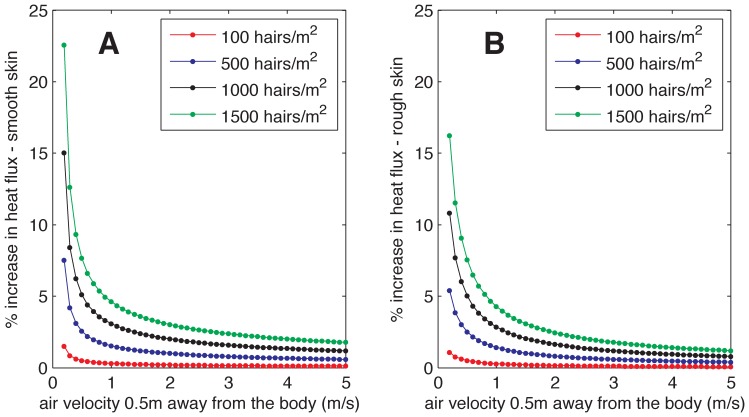
Increase in heat transfer due to hair, reported as a percentage *h_hair_*/*h_skin_*×100, as a function of ambient wind speed for different hair densities on smooth skin (A), and rough skin (B). The heat transfer increase due to hair on our example elephant – which has a hair density of ∼650 hairs/m^2^– is ∼10% at low wind speed and decreases as the wind speed increases.

The percent increases in heat flux due to the presence of hair, 100×*h_e,rough/_h_rough_* and 100×*h_e,smooth/_h_smooth_*, for a range of wind speed conditions (referenced at 50 cm from the elephant’s body) and hair densities, are reported in Figs. 4A and 4B. At the low density of 100 hairs/m^2^, equivalent to 0.01 hairs/cm^2^, there is little effect on heat transfer simply due to the small number of hairs. At higher, more realistic values, the heat transfer increases as the density increases, reaching up to 23% for smooth skin and up to 16% for rough skin when the hair density is about 1500 hairs/m^2^. This increase is highest under low-wind conditions when it would be most needed since the heat transfer coefficients would be lower (Fig. 3). The reduced effect of hair with increasing wind speed is related to a higher increase in the efficiency of heat transfer from the skin, compared to the lower increase in the efficiency of hair heat transfer (see Fig. 3).

The assumed wind speeds vary within a typical range expected at about 2 meters above the earth surface (for still elephants) or represent the relative wind velocities felt by the elephant when walking (at typical speed of 0.5–2.5 m/s [21]). Note that we do not model low wind speeds of less than 0.2 m/s because such very low wind speeds are infrequent in the environment and ear flapping by the elephant would increase the air flow past the body beyond 0.2 m/s. In addition, natural convection dominates at such very low wind speeds so our heat transfer model, which is based on forced-convection, would need to be modified.

This increased thermal exchange due to hair is not only important during the peak-temperature daytime hours. Recent work indicates that elephants have a capacity to store heat accumulated during the day through insolation and metabolic generation [12], and then to release this heat during nighttime (as evidenced by larger-than-expected Asian elephant internal temperature oscillations in outdoor conditions in Thailand compared to indoor conditions in Germany [12]). The heat dumping occurred in favorable nighttime climatic conditions allowing the elephants to begin the day with a thermal “deficit” – similar to desert dwelling animals like the camel – to accommodate an anticipated need for heat storage during the day. Therefore, even in conditions where the peak air temperatures exceed an elephant’s skin temperature, hair could help dump the stored heat during cooler nighttime hours, and enhance this “thermoregulation though heat storage” mechanism. This underlines the under-examined role of heat storage in the thermoregulation of elephants (and other large animals), which thus does not strictly mean maintaining a constant body temperature. Heat storage would be an important factor in large animals like elephants where the high volume to surface-area ratio also indicates high heat storage to heat flux capacity ratio.

## Discussion

These results demonstrate that elephant hair increases the effective heat transfer coefficient of the elephant significantly and is therefore a thermoregulation heat sink. Until now, to the best of our knowledge, there has been no example in nature of animal hairs increasing heat loss, let alone a beneficial reason for the phenomenon. Our work is consistent with findings for leaf hairs [22] and cactus spines [23], [24], whose sparse projections create an effect analogous to elephant hairs in both cases. It is likely that examples exist for other present-day biological organisms, particularly plants, which should be investigated in future work.

But to date, earlier workers noting the possibility of such an increase in animals remained skeptical whether the thermal-augmenting properties of hair were desirable [25]. However, the ability of epidermal structures to increase heat transfer ought to be expected [26]. At low densities, hair has almost no effect on air flow and does not trap an insulating air layer near the skin, but the extended hair acts as a pin fin that increases thermal exchanges with the surrounding air. Thus, as the hair density decreases from that of very furry animals, a break-even point is reached where the hair function switches from an insulator to a heat exchanger. This break-even point occurs at a density of about 0.3 million hairs/m^2^ [26] for thick hair covers with creeping flow in between (recall that 1500 hairs/m^2^ is about the maximum density of elephants). For comparison, the hair density of the human head is about 2 million hairs/m^2^ (see Methods and Discussion S1).

Elephants are the last remaining *Proboscidea* members. Current phylogenetic relationships within the order place them in the same family, *Elephantidae*, as woolly mammoths, *Mammuthus* [13], suggesting they share a recent common ancestor which might have had high, insulting hair densities. This relationship implies that the evolutionary residual nature of elephant hair is a possibility for its retained presence, but for today’s elephants which have been living in exclusively warm regions since before the last Ice Age [13], [27], [28], the question is: why have elephants retained any hair at all? Our study demonstrates that hair can help elephants transfer energy to their surroundings and hence suggests that retaining low density hair covers could be the result of the evolutionary advantages such a cover provides.

Future work could shed more light on this possibility by evaluating whether large (extinct) animals with similar disadvantageous body-volume to surface-area ratios as the elephant’s had such coverings. More generally, the heat transfer effect of elephant hair challenges the belief that a sparse hair layer would have provided insulation early on in its evolutionary development. Since proto-hairs are believed to have developed from single barbless shafts [29] (similar to elephant hair) that increased in density over time, our results are cause for further investigation as to whether hair could have initially evolved to increase, instead of decrease, heat transfer.

## Supporting Information

Methods and Discussion S1 **Further details on the hair counting approach and algorithms and on the heat transfer model and its sensitivity.**
(PDF)
