# Supplementary material for: What Is the Use of Elephant Hair?
Source: PLoS One. 2012 Oct 10;7(10):e47018. doi: 10.1371/journal.pone.0047018 (PMC3468452; doi:10.1371/journal.pone.0047018)
Supplement: Methods and Discussion S1 — Further details on the hair counting approach and algorithms and on the heat transfer model and its sensitivity. (PDF) [file pone.0047018.s001.pdf]

# Supporting Methods and Discussion for "What is the Use of Elephant Hair?"

**Conor L. Myhrvold[1] , Howard A. Stone[2] , Elie Bou-Zeid[1*]**

[1] Department of Civil and Environmental Engineering, Princeton University, Princeton, New Jersey, United States of America

[2] Department of Mechanical and Aerospace Engineering, Princeton University, Princeton, New Jersey, United States of America

[*] Email: ebouzeid@princeton.edu

This document contains supplementary material and details of the methods that were used in the paper.

## Pin Fin Assumption

There are several reasons why elephant hairs can function as pin fins. First, hair reaches colder and faster air away from the skin, thus penetrating outside of the skin boundary layer, creating larger temperature differences with the air, and increasing heat transfer [1], [2]. Since the thermal conductivity of mammal hair and skin (0.37 – 0.475 W/(mK), Table 1 in main text) is ~14-18 times that of air (0.0263 W/(mK) at 30 °C), hairs transfer heat from the skin faster than conduction in air. Hairs also add to the animal's total surface area similar to an epidermal crack or crease; since all of these features are evident in elephants [3], there is an increase in the total elephant skin area available for heat transfer. If one assumes that the length of the hair contributing to heat exchange is 20 mm and its diameter is 0.5 mm, this increase in area for 1500 hairs/m$^2$ is 4.7%. In practice, not all 20 mm contribute to heat exchange as discussed in the main text.

## Elephant Hair Parameters

In the absence of quantitative data we used a range of hair densities, lengths and diameters that are in-line with other mammals (such as mammoths) and qualitative observations of elephants as described in the literature over the past few decades.

- Elephant hairs can reach up to 76 cm in length and 3.5 mm in width [4]. However, these maximum hair length and diameter values are exclusively for the tail and thus are of limited use for us; we need body hair parameters.
- Elephants and mammoths are shown to have a nearly identical hair structure [5]. Mammoth guard hair has a diameter of 0.25 – 0.5 mm and therefore is another metric (but other "epidermal protuberances" of 0.3 – 0.6 mm diameter also exist) (Mammoths: Giants of the Ice Age. by Adrian Lister, Paul G. Bahn).˙ From an evolutionary standpoint, mammoths and elephants are closely related. It is not clear what type of hair modern-day elephants inherited from mammoths, or if current body hair is altogether a distinct combination.
- Another source gives an Asian elephant hair diameter as 0.3 mm (309 micrometers) (http://web.me.com/kwpmiller/HAIRbase/Asian_Elephant/Asian_Elephant.html)).
- The hair diameter has much more of an effect on our results (see discussion of Fig. S2 below) than the hair length. This is because we care not about the absolute length of the hair but rather the length over which heat exchange takes place, which can be substantially shorter if there is a high temperature gradient close to the skin surface; recall that in our model results only the lower third of the hair contributes significantly to heat exchange.

## The Role of Hairs in Extremely Hot Conditions

When the temperature of the ambient environment is higher than that of an elephant's body temperature, increasing an elephant's heat transfer coefficient through the mechanism examined in this study would cause an elephant to gain more heat through the shaft and the base of body hairs, which facilitates a higher heat transfer rate than the air in the vicinity. However, heat gain from solar irradiance is often of greater concern that the convective heat gain from air during such conditions.

Thus a commonly observed strategy for elephants under extreme heat is behavioral; either cooling off in water, "dusting" by throwing dirt on their backs, or moving into the shade. Dusting reduces the solar radiation load of the elephant by greatly increasing the reflectivity of its skin in the near infrared range, since in that range the reflectivity of dry dirt and dust does not have the water absorption bands that elephant skin has. However, this behavior comes at the expense of convective heat transport through the hairs and skin, which is decreased as the elephant body surface is insulated by a layer of dirt and dust.

Moving into the shade of trees would help elephants reduce their radiative gains and make their net radiative exchange negative; this radiative cooling is very helpful. However, since elephants are highly nomadic and diurnal, they need to frequently travel in the open in savanna and desert environments where little shade exists. Shade (and, for that matter, access to water) is often not an option. In such cases, it seems that elephants are able to store heat, i.e. to temporarily increase their body temperature, which they can later release either at night by convection and radiation or when they gain access to water [6].

## Wind Speed Computation for the Heat Transfer Model

All of the heat transfer rates computed in this paper are functions of the ambient wind speed, and how it is reduced as a function of the distance from the elephant skin. For the ambient wind speed variability, we use a range of 0.2 m/s to 5 m/s, which is representative of expected wind speed near the earth surface. The variation of the wind speed with distance from the skin is then computed based on (1) this ambient wind speed, assumed to occur at a reference distance of 0.5 m away from the body, and (2) the distance from the skin, via well-established formulations for wall-bounded, steady-state flows [8]. While these formulations strictly apply for sufficiently large distances from the "edge" of the body, i.e. after the flow had equilibrated with the skin drag, they provide a reasonable estimate of the actual wind speed near the skin since this equilibrium is reached rapidly when the incoming flow is turbulent, which is the case for air flow approaching the elephant skin. In addition, taking into account the adjustment zone or choosing a different reference distance would have similar effects on our results as a moderate change in the ambient wind speed range, and thus would not affect our conclusions.

Away from the skin, the formulation we use is the so-called log-law given by:

$$u(z) = \frac{u_*}{\kappa} ln\left(\frac{z}{z_0}\right), \qquad (1)$$

where $u$ is the air speed at a distance $z$ from the skin; $\kappa$ = 0.41 is the von Karman constant; $u_*$ is the so-called friction velocity, which is the square root of the kinematic stress at the skin (force per unit area from the wind on the skin divided by the air density). This relationship applies in the log-layer which extends from $z = 25\ \nu / u_*$ to $z$ = 0.5 m for the smooth skin, and from $z = 2\ z_r$ to $z$ = 0.5 m for the rough skin, where $\nu = 1.603\times10^{-5}$ m$^2$/s is the kinematic viscosity of the air and $z_r$ is the roughness of the elephant skin (≈ 5 mm in this paper). For the rough elephant skin,

$z_{0,rough} = z_r /10 = 0.5$ mm [9]. For the smooth skin, the roughness length depends on the air viscosity, rather than the roughness features, and is given by $z_{0,smooth} = \nu / (8.4\ u_*)$ [8].

Very close to the surface over the smooth skin, in the so-called viscous sublayer where $z < 5\ \nu/ u_*$, the velocity profile is given by [8]:

$$u(z) = u_* \left( \frac{z u_*}{\nu} \right). \tag{2}$$

For the rough skin, close to the surface, the dynamics are dominated by the skin roughness elements and the velocity profile depends on the properties of these elements (rather than viscosity). One simple model for the roughness element layer ($z < z_r$), which is in essence analogous to the approach used for the viscous sublayer yielding equation 2, is to assume that the non-dimensional velocity $u(z)/u_*$ is proportional to the non-dimensional height $z/z_r$, where the roughness elements scale $z_r$ is used as the mixing length in that layer. We use this linear model for the roughness sublayer, with the same $u_*$ determined from the log-law outside the roughness layer (it should be the same since it is related to the stress at the skin-air interface) and with the proportionality constant, $\eta = 3.65$, determined such as to match the log-law velocity profile (equation 1) at $z = 2\ z_r$ :

$$u(z) = \eta u_* \left( \frac{z}{z_r} \right). \tag{3}$$

The friction velocity is computed first from equation 1 based on the imposed wind speed $u(z)$ at $z = 0.5$ m from the body using the roughness length $z_0 = 0.5$ mm for the rough skin, or iteratively for the smooth skin since $z_0$ also depends on the friction velocity. Then, the wind speed can be computed for the smooth skin for $zu_*/\nu < 5$ from equation 2 and for $zu_*/\nu > 25$ from equation 1, and for the rough skin for $z < z_r$ from equation 3 and for $z > 2\ z_r$ from equation 1. For

$5 < zu_*/\nu < 25$ (the so-called buffer layer) for the smooth wall, and for $z_r < z < 2\, z_r$ (the so called roughness sublayer) for the rough wall, the velocity is interpolated using cubic splines from the velocity profiles below and above that range. This approach implies that the friction velocity and variation of air velocity with distance $z$ are already jointly determined by our imposed wind speed at $z$ = 0.5 m and also, for the rough wall only, by the roughness element scale $z_r$. An example of the velocity profiles obtained used this approach for the reference wind speed $u$(0.5 m) = 0.5 m/s is depicted in Fig. S1, along with some illustrative heights. As expected, the smooth skin has higher velocities than the rough skin.

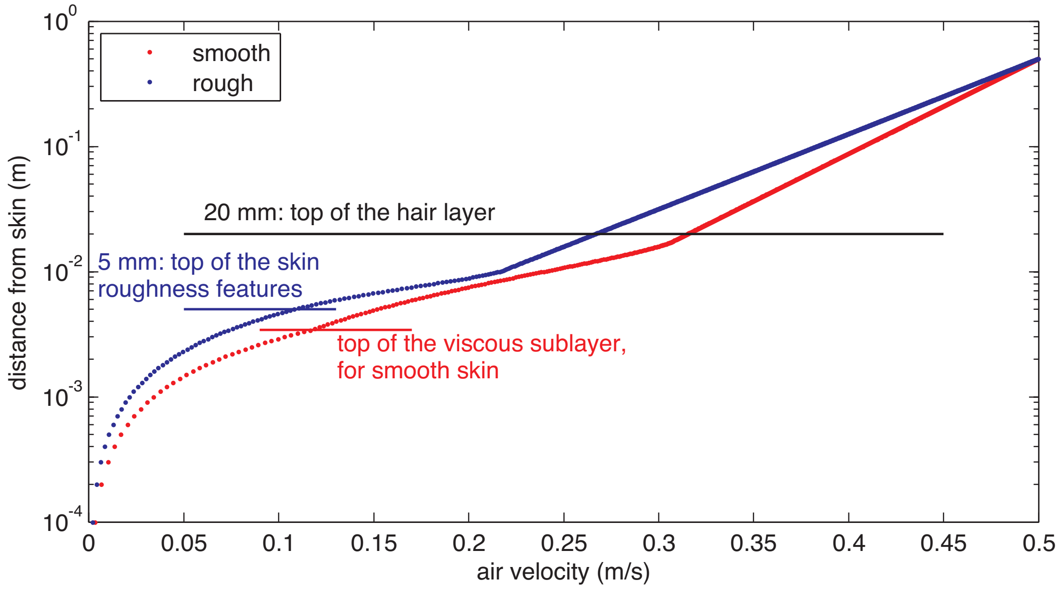


**Fig. S1. Velocity profiles for the smooth and rough skins,** also depicting the top of the viscous sublayer, the top of the roughness element layer, and the top of the hair layer.

## Hair Diameter Effect

Fig. S2 illustrates the effect of hair diameter at various wind speeds. The assumed hair density is constant at 1500 hairs/m$^2$. The variation of the heat transfer from the hair is nearly proportional to the hair diameter and the increase in heat transfer due to hair remains significant for thinner hairs at low wind speeds.

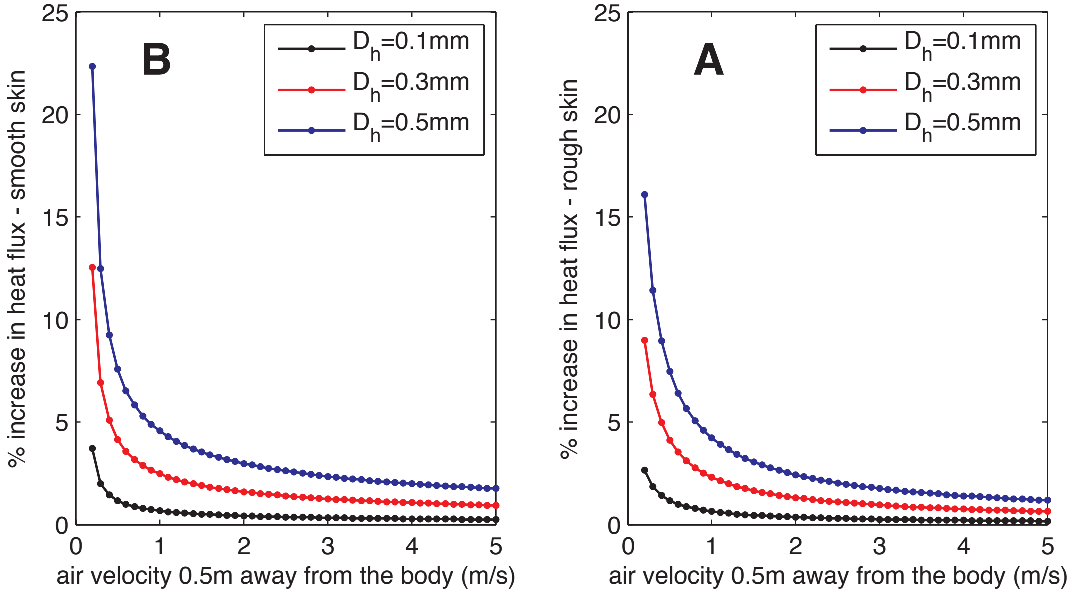


**Fig. S2. Effect of hair diameter.** Variation of the increase in heat transfer due to hair with hair diameter, and wind speed, for a hair density of 1500 hairs/m$^2$.

## Extended Technical Discussion of Previous Studies on the Role of Ears

As mentioned in the main text, prior thermoregulatory assessment of ear flapping and vasodilation used infrared thermography and thermal modeling based on elephant ear parameters. The magnitude of the effects, expressed as a percentage of the heat flux that would match an elephant's estimated heat transfer needs, are extremely varied (from 8% [7] to 100% [10], [4], owing to the fact that they use significantly different testing scenarios). Studies suggesting higher contributions of up to 100% SMR [4] , [10] are based on an extremely limiting set of modeling assumptions and ambient conditions, possibly unrealistic for an elephant's typical environment. The two main papers are by Phillips and Heath [4]  and Williams [7], both of which considered captive African elephants.

Both papers calculate longwave radiative heat losses to the atmosphere, accounting for radiative gains from the atmosphere. While interesting from an engineering perspective, from an elephant evolutionary perspective these calculations are not always relevant since one would also need to account for shortwave radiative gains of the elephants from direct and diffuse solar radiation for elephants living in the wild. Both studies neglect shortwave radiation yet this source is more important than longwave radiation during daytime, where the elephant heat dumping needs are at a maximum (Philips and Heath note that their analysis would not be relevant under "periods of drought or intense solar radiation").

Phillips and Heath compute only a maximum of about 5% of the metabolic rate that is lost through the ears, but then they suggest, according to a model of heat transfer that they construct, that under a 5 m/s wind (a rather strong wind) and 20 °C air temp (cooler than many elephant climates) the loss can be 91%. However, under these conditions the elephant can

probably lose enough through other parts of the body, which comprise 80% of an elephant's surface area. Williams on the other hand performed his study under an ambient temperature of 12.6 °C, a much cooler environment that the elephant's natural habitat. Thus, while both studies suggest that an important role of the ears is possible, neither confirms that heat loss from African elephant ears is nearly enough to meet the elephant's heat dumping needs.

Infrared thermography has since been applied to captive Asian elephants by Weissenbock *et al.* [11], [12]. This subsequent work confirms that Asian elephants experience similar skin surface temperature gradients on the ears and other body locations, but remains inconclusive as to whether the ears can account for all of the required heat loss.

Furthermore, ear flapping can be a behavioral communicator (when elephants are excited or irritated) and other authors have argued that flapping is primarily performed for communication and not cooling [13].

Finally, and very importantly for this study, the fact that elephant ears are covered with hair means that any increase in the heat transfer rate due to hair on the body will also affect the ears and thus an important role of ear flapping in thermoregulation is not contradictory to the effect of body hair on thermoregulation.

## Determining Elephant Hair Density

We determined the hair density of African elephants using photographs analyzed with image-processing routines in Matlab™. The dataset we have at present is based on 3 photographs of Sherini, a 24 yr female elephant in Botswana who stands 232 cm tall with a rear foot length of 40 cm, one of her leg, ear, and the back of leg (Fig. S3). The photographs were provided by Dr. Kate Evans of ElephantsForAfrica (photos were taken in Botswana on captive elephants used in the safari industry with permission from the owners and from the Office of the President of Botswana and the Department of Wildlife and National Parks (permit number EWT 8/36/4XVIII(50)). Each photo, at the time of taking, contained a ruler placed against the elephant skin to get a pixel to cm conversion in each of the photographs (Supplementary Table 1), determining how much real-world space a given distance on the photograph spans, in terms of pixels.

Next, we cropped the photos and marked hair locations in each one in Photoshop™ CS5 using a 6-pixel diameter dot from a red paint brush with 100% hardness (Fig. S3). Each hair location is a dot. We then took those photographs and put them back into Matlab™. A script uses image processing to recognize and isolate the red dots from the background, determining the x-y coordinate of each dot and thus the hair (Fig. S4).

One way to compute a density is to determine the distance between each nearest neighbor hair. To do this we use Delaunay triangulation, a computational geometry method, which constructs a triangular mesh using the data points. The distance from one hair to its nearest neighbors is the length of the triangle vertices (Fig. S5). Afterward, we filter out the outliers inherent in the Delaunay process by using histograms, which is one way of inferring

uncertainties (Figs. S6, S7, S8).

For each photograph we compute the mean, standard deviation, and variance for each of the points (hairs) in the triangulation and its nearby neighbors (Supplementary Table 1). We test whether the resulting mesh represents a Poisson or a log-normal distribution (Figs. S9, S10, S11). We use the histograms to compute a range of the possible densities from the data and get a measure of the variability of the hair distributions observed in the photographs. We are also able to get the hair density from knowing the total hair count (a robust sample size of several hundred for each image) and the pixel area of these particular photographs (Supplementary Table 1).

The hair density does not model like a Poisson disk or log-normal distribution. The hair density distribution is uniformly distinct from the two modeled distributions in both cases, and it is not random. On three sections of Sherini's body, the hair density was between 0.03 – 0.07 hairs/cm$^2$.

Asian elephant hair densities are undoubtedly higher than their African counterparts. Additionally, visual observations of African elephants indicate that hair density varies across the body. Finally, a careful scrutiny of elephant photographs reveals a source of uncertainty that leads to an undercounting of hairs. The camera resolution, and thus the ability to detect hairs, is the current limitation of the method, and is likely to be the largest source of uncertainty. Therefore, we investigate a realistically wider range of hair densities in our model.

## Matlab™ Analysis of Hair Density (in the Order of Operations)

**Pixel-to-cm conversion: taking measurements.** Each photo was loaded into Matlab™. The imread and ginput functions were used to take 1 cm measurements along the ruler in each of the photos, fitting the images to an x-y coordinate system. We clicked multiple times in each photo to make as many measurements as possible to account for clicking error.

**Photoshop™ edits.** The images were originally edited in Photoshop™ (curves adjusted, sharpened, etc.) to best see the hairs. A Photoshop™ mask was used to preserve the ruler readings while at the same time optimizing the image exposure to be able to see and count hairs. We adjusted the exposure to see the black hairs on the gray skin. They were then cropped to the final hair counting areas, and red dots are placed on perceived hair locations. We added red dots to enhance the ability to detect the hairs in Matlab™.

*Table A.* **Pixel-to-cm conversion: getting the numbers.** The data had been assembled separately for the x and y coordinates. These matrices were then combined and associated with the individual image names. Since the 1 cm distances were determined pairwise (clicks 1&2 and 3&4 representing two distinct measurements), we computed the distance between each pair to get the pixels per 1 cm on the ruler. Because the rulers were at a slight angle, we computed the Euclidean distance difference instead of just the difference in the x-direction (although in theory the rulers were all placed horizontally, as is evident in the original photographs, Fig. S3). These results indicate that the ear and leg photographs were taken at the same distance away from the skin. We used the mean due to the low standard deviations in comparison to the measurements.

**Mapping the hair densities in Matlab™.** We loaded the images again into Matlab™ and eliminated all the features except the red points. We started off with a color image of an elephant (Fig. S3*)*. Color images are composed of 3 channels: red, green, and blue so we manipulate the threshold data in 2 of the channels, one of which clearly shows our red dots and

the other which clearly does not (a background and a foreground). We subtract one from the other yielding a black-and-white thresholded image (Fig. S4B). However, that image still has some noisy specks, so then we use another function with a size threshold (bwareaopen) to filter out the small specks from the dots (Fig. S4C). We can use a specific size threshold because we standardized the dots to have a diameter of 6 pixels in Photoshop™, so we know what the maximum pixel threshold is before the script would filter out hair locations (which we would not want to have happen). Then we use bwlabel which looks for white pixels surrounded by other white pixels; all 8 neighboring pixels have to be white. Bwlabel returns the coordinates of those coordinates and we take the average to get the center of each dot, the hair location (Fig. S4D).

*Table B.* **Hair map statistics.** The density measurement (hairs per unit area) is intrinsically a statistical quantity that averages the density at each hair. Each hair is surrounded by its nearest neighbors. The density estimate at each hair site is the inverse of the average distance to its nearest neighbors. To get the distances to the neighbors we could calculate the distance from each hair to every other hair, then sort to find the nearest ones. That would work, but for N hairs there would be $N^2$ distances, the vast majority of which are not interesting to us because the hair density only relies on nearest neighbors. Delaunay triangulation is an algorithm in computational geometry that, given a set of points, finds the nearest neighbor points to each point, so it is a computationally more efficient alternative (it is also a built in function in Matlab™). We then calculate the mean, standard deviation (std dev), and variance (var) of those sets of distances, in addition to the std dev/mean and the var/mean.

*Table C.* **Filtering outliers.** However, there is an important caveat attached to these values. By definition, there will be outliers which skew the above statistical measures of distances between nearest neighbors: if you have a finite mesh you will always have edge points. Those points will not be surrounded by other points on all other sides because they are located on

the edge. Consequently, we remove edges by filtering out the data and imposing a cutoff to get rid of (most of) the edge influence by restricting the data to distances under 125 (determined by viewing the histograms, which are explained below). After the cutoff is imposed, the statistics change, as seen in Panel C of Figs. S6, S7, S8.

**Histograms.** We create a histogram and figures to show what the triangulation looks like. Since hair density is the inverse of inter-hair distance, each of these histograms could be interpreted as a histogram of hair density measurements by taking the inverse of the peak of the distribution (1 over the value of the highest bin on the histograms). However, we opt to directly measure hair density by counting the numbers in the image of the photographed skin patch. All hairs are not spaced to the common spacing, which the mean of the distances using Delaunay triangulation would indicate.

**Distribution testing**. We then fit a Poisson distribution with the same mean and variance over what we actually observe for elephant hair densities, to see if they are similar (they are not). The observed variance (y-axis) is too high, and the data "tail off" more quickly (i.e. the slope is more steep) than a Poisson distribution. Finally, we fit a log normal distribution using Matlab™'s lognfit function to see how the elephant density values compared with another possible distribution. Note that we do not use the actual probability distribution function, as the odds of getting any particular value are low. Instead we look at the cumulative distribution function of the Poisson distribution, and take the difference between the successive points, which is an equivalent to the sum of the probabilities of getting anything between those two points. The elephant hair distribution across our 3 photographs is not consistent with a log normal distribution either.

*Table D.* **Densities.** Finally, we get the hair density values by taking the total area of the images and counting the number of observed hairs in each image. These values are used as an anchor point for the range of hair densities used in our model calculations.

## Comparison to Other Mammalian Hair Densities

The elephant hair density range of Sherini, our example elephant, is 0.03 – 0.07 hairs/$cm^2$. Chinchillas have the densest mammal body hair, apparently 20,000+ hairs/$cm^2$ (http://www.chinchillaplanet.com/chinchilla-facts/). In comparison, human heads are ~200-300 hairs/$cm^2$ (http://www.keratin.com/aa/aa014.shtml). Rabbit backs are 4,100±260 hairs/$cm^2$ (sample size of 5 rabbits), horse flanks 1,290±140 and bellies 850±90 hairs/$cm^2$ (sample sizes of 8 and 3 horses), and pig flanks 37±5 and bellies 8.5±2.4 hairs/$cm^2$ (sample sizes of 4 pigs in both instances) [14]. Since we know other parts of an African elephant are hairier, in addition to the fact that Asian elephants are hairier than Africans, the hair density range estimate serves as a conservative baseline that can be expected to vary upwards, to a density several times greater (hence the hair densities used in our models). Therefore, our hair density estimate from three locations of an individual elephant is in agreement with prior published hair density estimates, so we believe it represents an adequate estimate of hair density.

## Supplementary Table 1.

**A. Pixel-to-cm Conversion.**

| Image | Measurements (1 cm intervals) | Pixels/cm (mean) | Std dev (pixels) |
|---|---|---|---|
| Back of Leg | 9 | 102 | 3.89 |
| Ear | 16 | 92.6 | 6.66 |
| Leg | 6 | 91.7 | 1.37 |

**B. Before Cutoff.**

| Image | Mean | Std dev | Var | Std dev / mean | Var / mean |
|---|---|---|---|---|---|
| Back of Leg | 49.7 | 33.3 | 1111 | 0.670 | 22.3 |
| Ear | 62.9 | 60.3 | 3632 | 0.960 | 57.7 |
| Leg | 67.4 | 46.5 | 2166 | 0.690 | 32.1 |

**C. After Cutoff.**

| Image | Mean | Std dev | Var | Std dev / mean | Var / mean |
|---|---|---|---|---|---|
| Back of Leg | 45.84 | 15.81 | 250 | 0.34 | 5.45 |
| Ear | 54.53 | 19.04 | 362 | 0.35 | 6.64 |
| Leg | 60.93 | 20.12 | 405 | 0.33 | 6.65 |

**D. Hair Density Calculation.**

| Image | Pixels/cm | Image Area (widthxheight, pixels) | Area (pixels) | Area ($cm^2$) | Hair Count | Density (Hairs/$cm^2$) |
|---|---|---|---|---|---|---|
| Back of Leg | 102 | 1108x662 | 733496 | 7191 | 461 | 0.0641 |
| Ear | 92.6 | 1103x838 | 924314 | 9982 | 401 | 0.0402 |
| Leg | 91.7 | 1085x866 | 939610 | 10247 | 325 | 0.0317 |

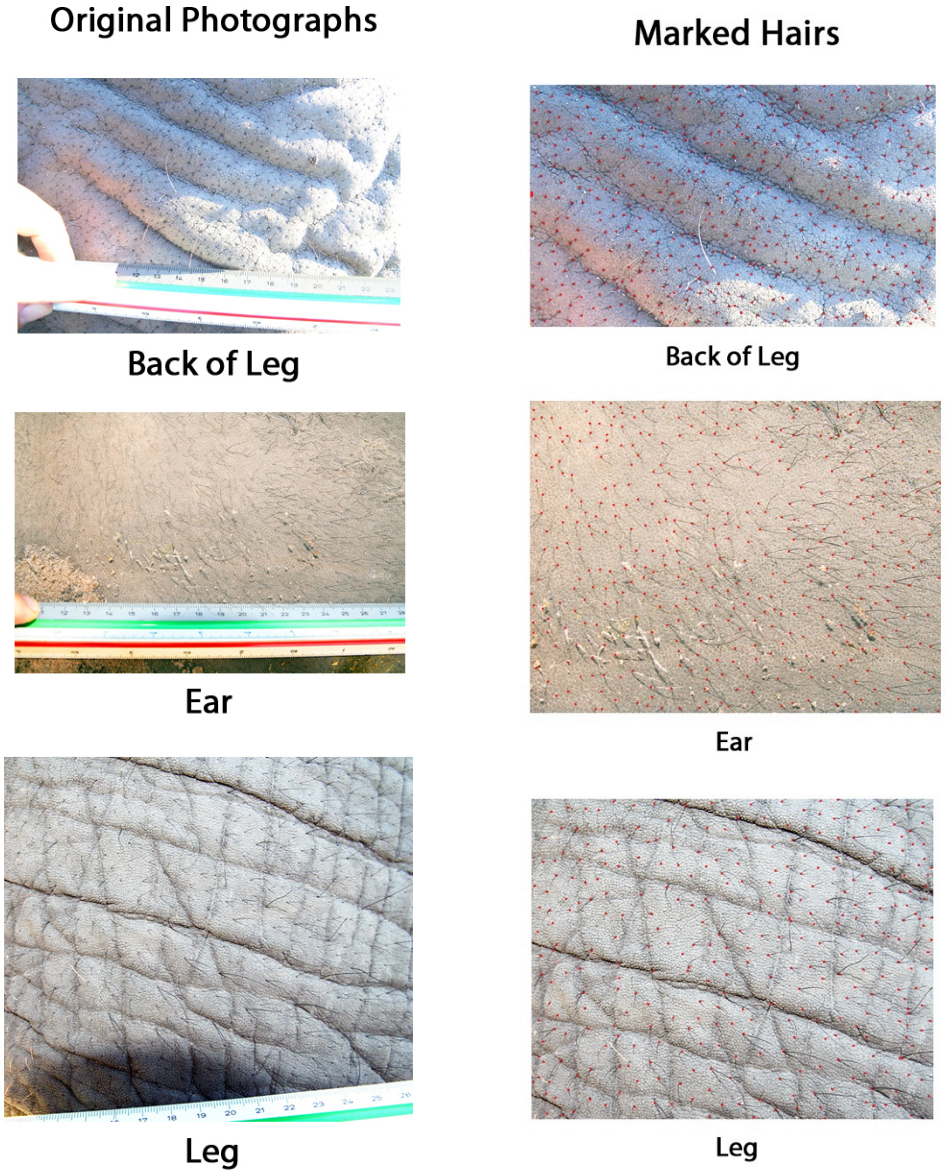


**Figure S3.** Pictures of different parts of Sherini (left) with scale, and enlarged view with the hair locations marked with red dots (right).

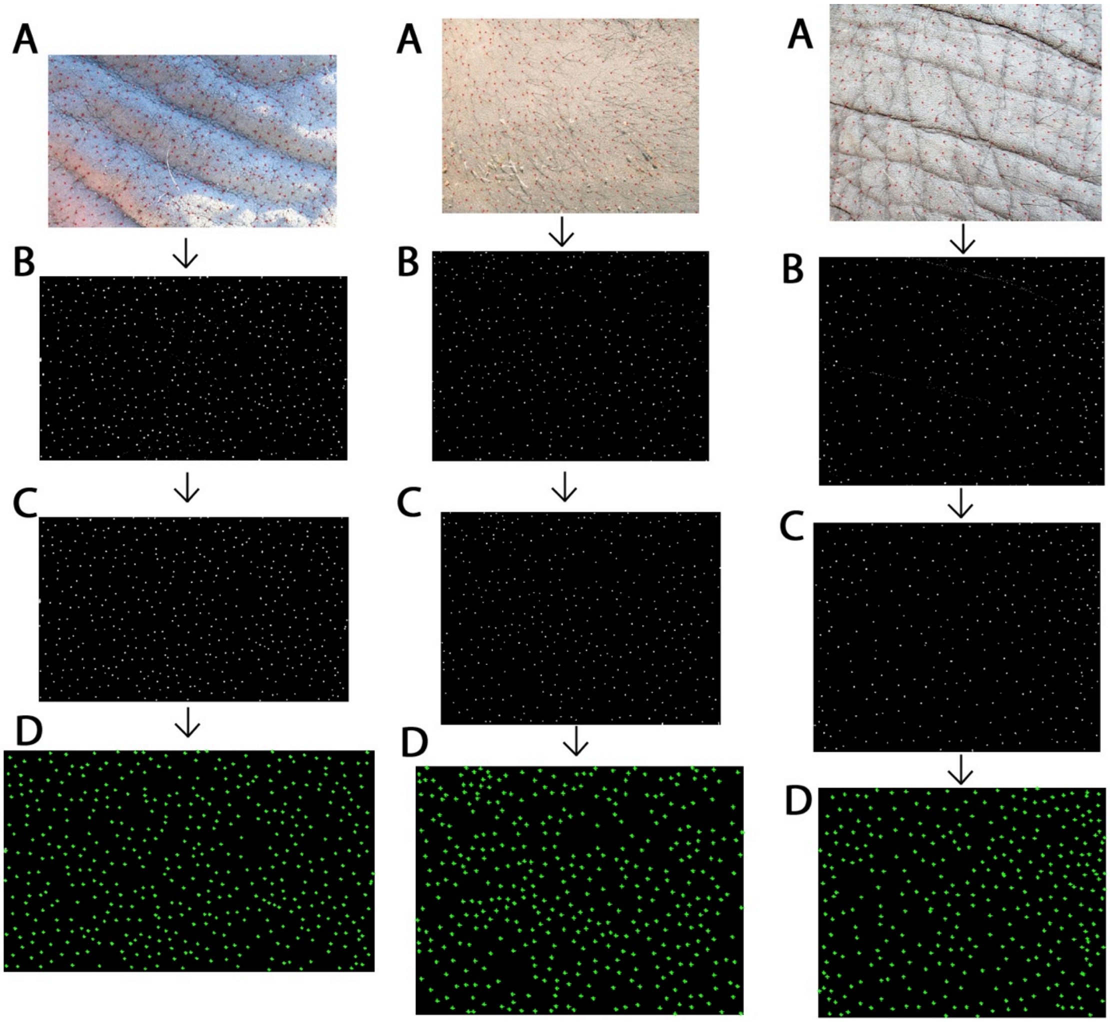


**Figure S4.** The original image (*A*) is converted into one where the dots are visible, along with other background artifacts (*B)*, which are then taken away (*C*). The remaining dots, which are the hair locations, are mapped onto a coordinate system (*D*). The mean pixel location – the middle of the dot – is the new (1 pixel) coordinate location.

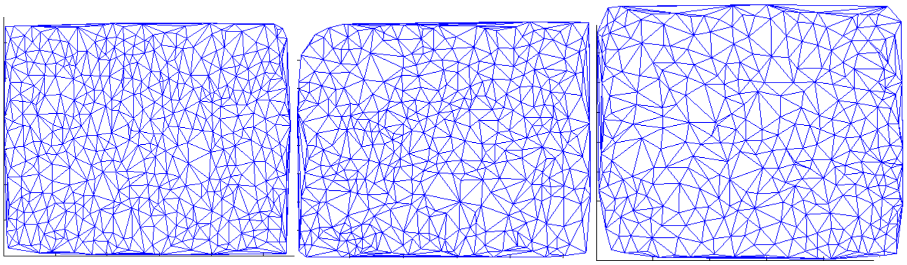

**A** **B** **C**

**Figure S5. The Delaunay triangular mesh.** Each vertex is a hair location. Any finite patch will have edges, and we need to eliminate those. We can do that by simply cutting off any distance greater than 125. Note how the edges are "squeezed" because we have finite sample of the hair. In reality, there are hairs on the areas surrounding the patch that we are examining. These edge effects would bias the quantitative statistical measures because they disproportionately "weight" the perimeter of the image. This can be seen in a histogram of the distances (Fig. S6 for Sherini Back of Leg, Fig. S7 for Sherini Ear, Fig. S8 for Sherini Leg,).

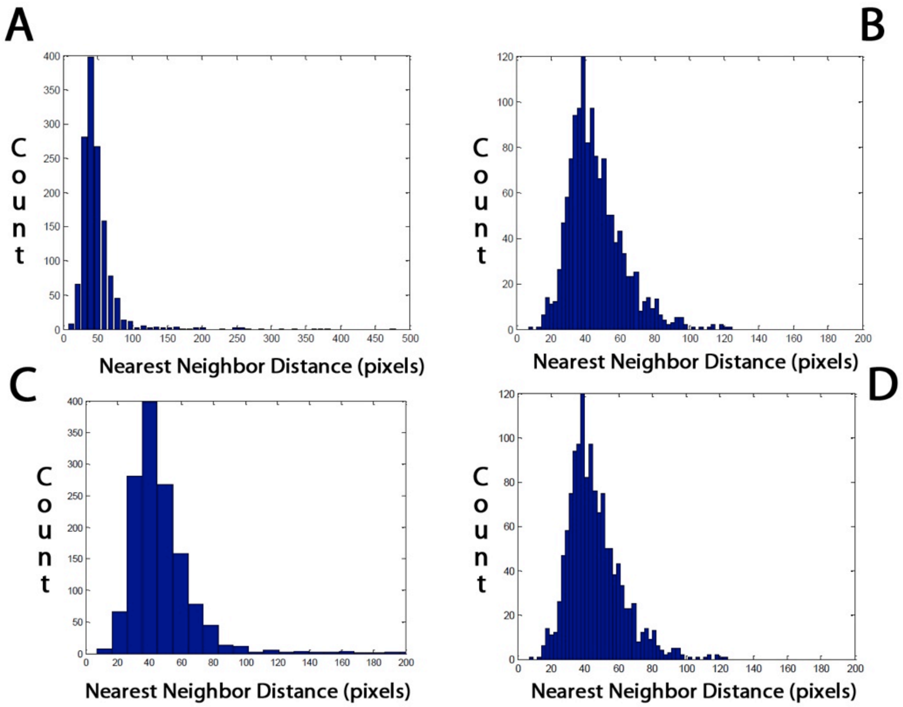


**Figure S6.** Back of Leg: ***A***: A histogram of the distances between the intersections on the mesh. The outliers on the right are from the edges. The distribution is largely Gaussian distribution. ***B***: One could use more computational geometry methods to remove the edge hairs. However it is simpler to impose the cutoff to the right of the hump of the distribution. ***C***: We can ignore the outliers visually quite easily: we "zoom in" and restrict the x-axis to only display the main distribution. It tends to be weighted to the right; otherwise it looks like a normal distribution. ***D***: with the imposed cutoff, and a finer resolution (more bins). In this example the distances range from 20-80 pixels.

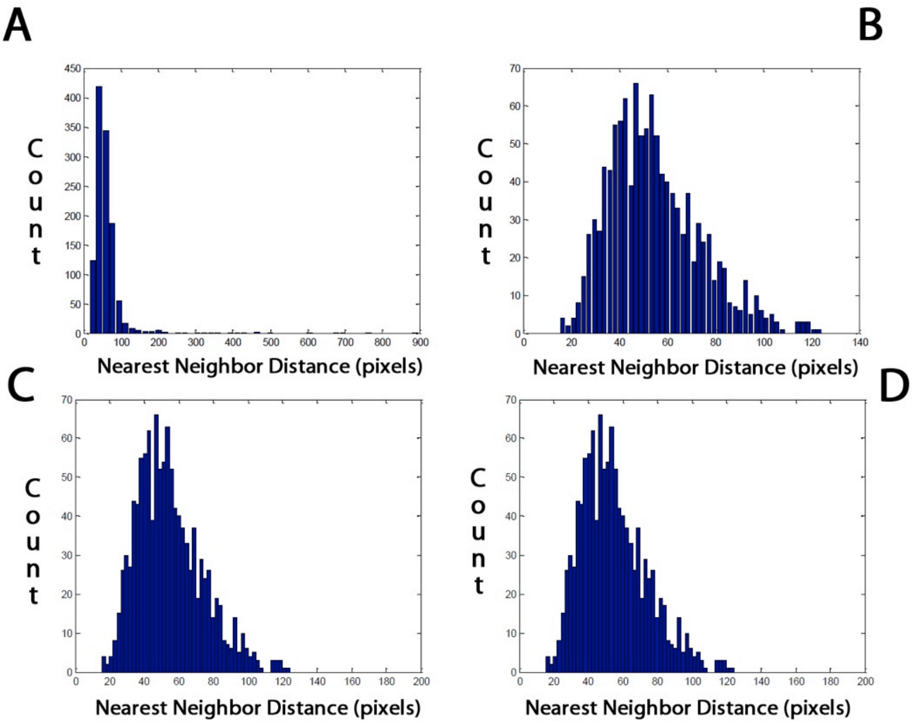


**Figure S7.** Ear: ***A***: A histogram of the distances between the intersections on the mesh. The outliers on the right are from the edges. The distribution is largely Gaussian distribution. ***B***: One could use more computational geometry methods to remove the edge hairs. However it is simpler to impose the cutoff to the right of the hump of the distribution. ***C***: We can ignore the outliers visually quite easily: we "zoom in" and restrict the x-axis to only display the main distribution. It tends to be weighted to the right; otherwise it looks like a normal distribution. ***D***: with the imposed cutoff, and a finer resolution (more bins). In this example the distances range from 25-105.

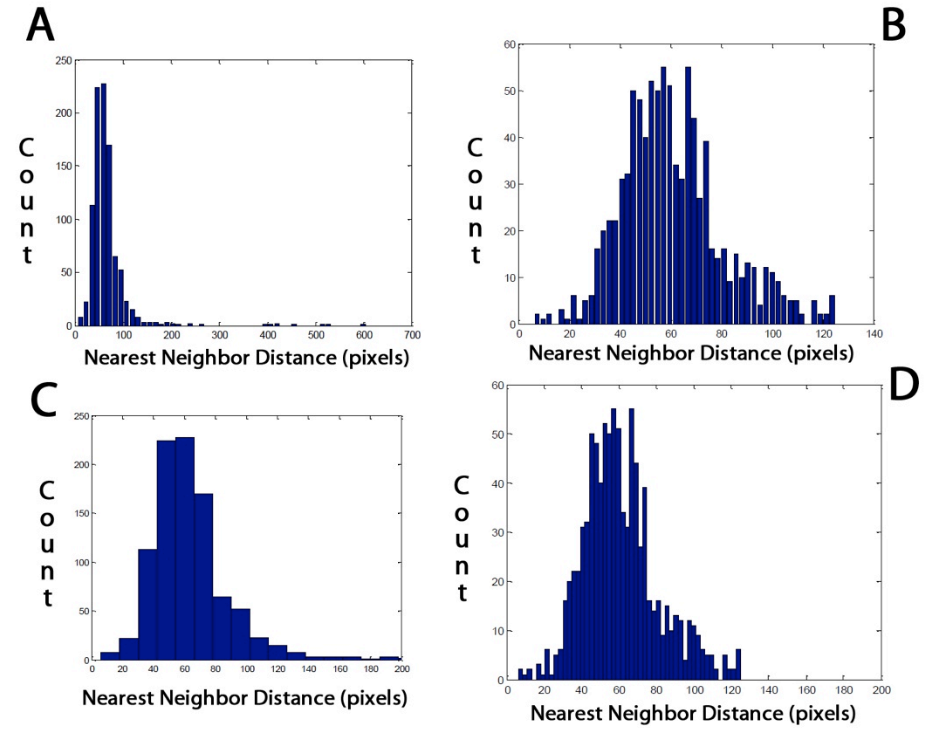


**Figure S8.** <u>Leg: </u>***A***: A histogram of the distances between the intersections on the mesh. The outliers on the right are from the edges. The distribution is largely Gaussian distribution. ***B***: One could use more computational geometry methods to remove the edge hairs. However it is simpler to impose the cutoff to the right of the hump of the distribution. ***C***: We can ignore the outliers visually quite easily: we "zoom in" and restrict the x-axis to only display the main distribution. It tends to be weighted to the right; otherwise it looks like a normal distribution. ***D***: with the imposed cutoff, and a finer resolution (more bins). In this example distances range from 35-125 pixels.

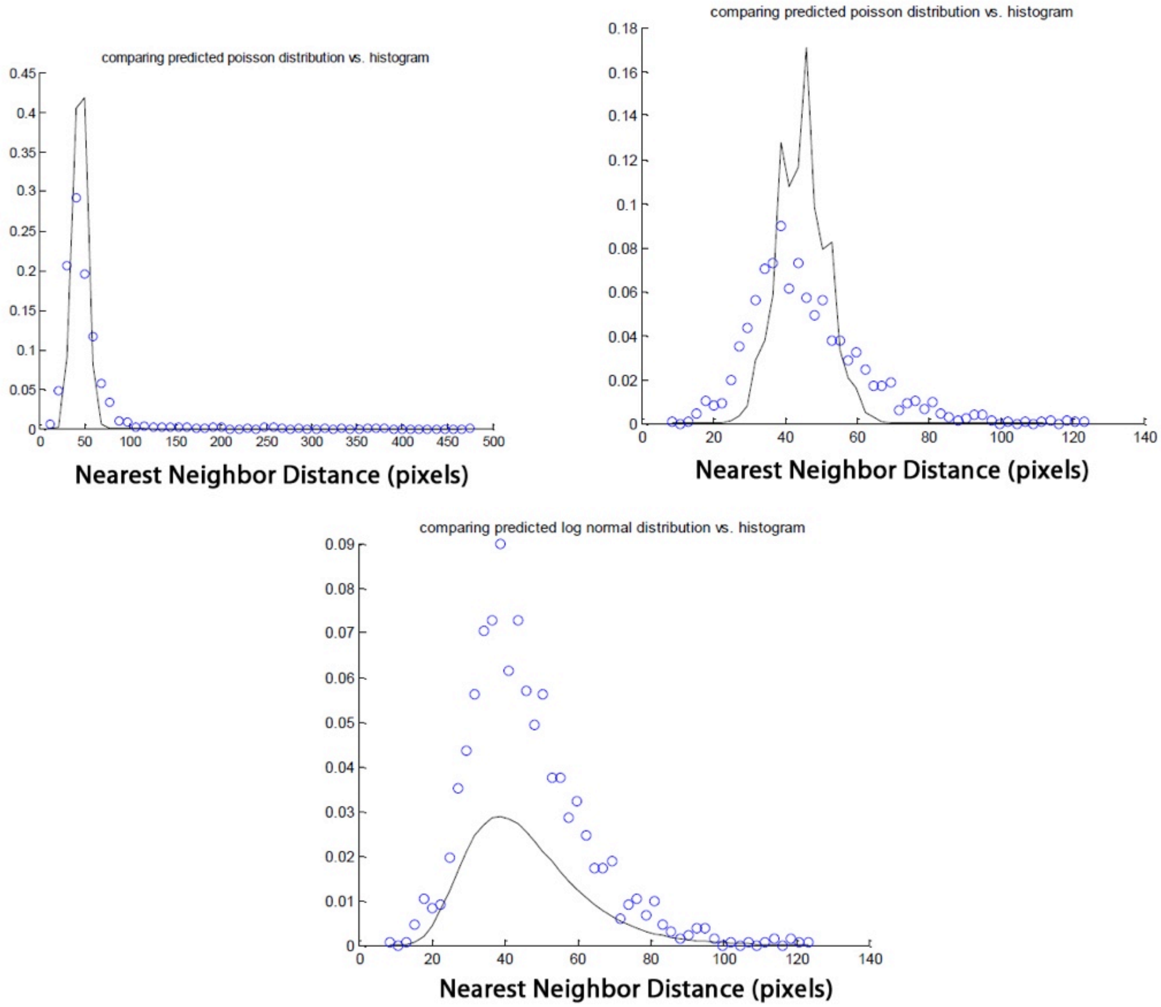


**Figure S9.** Sherini Back of Leg: *Above left*: The histogram (black line) is fit to a Poisson distribution (blue circles). *Above right*: the new Poisson distribution with the imposed 125 distance cutoff. Note that in reality, the mean is to the right of what we would expect if elephant hair density was like a Poisson distribution. *Below*: The histogram (black line) is fit to a log normal distribution (blue circles). However, the observed elephant hair density variance in this case is too small. This is the opposite of the Poisson case where the observed variance was higher than the modeled variance.

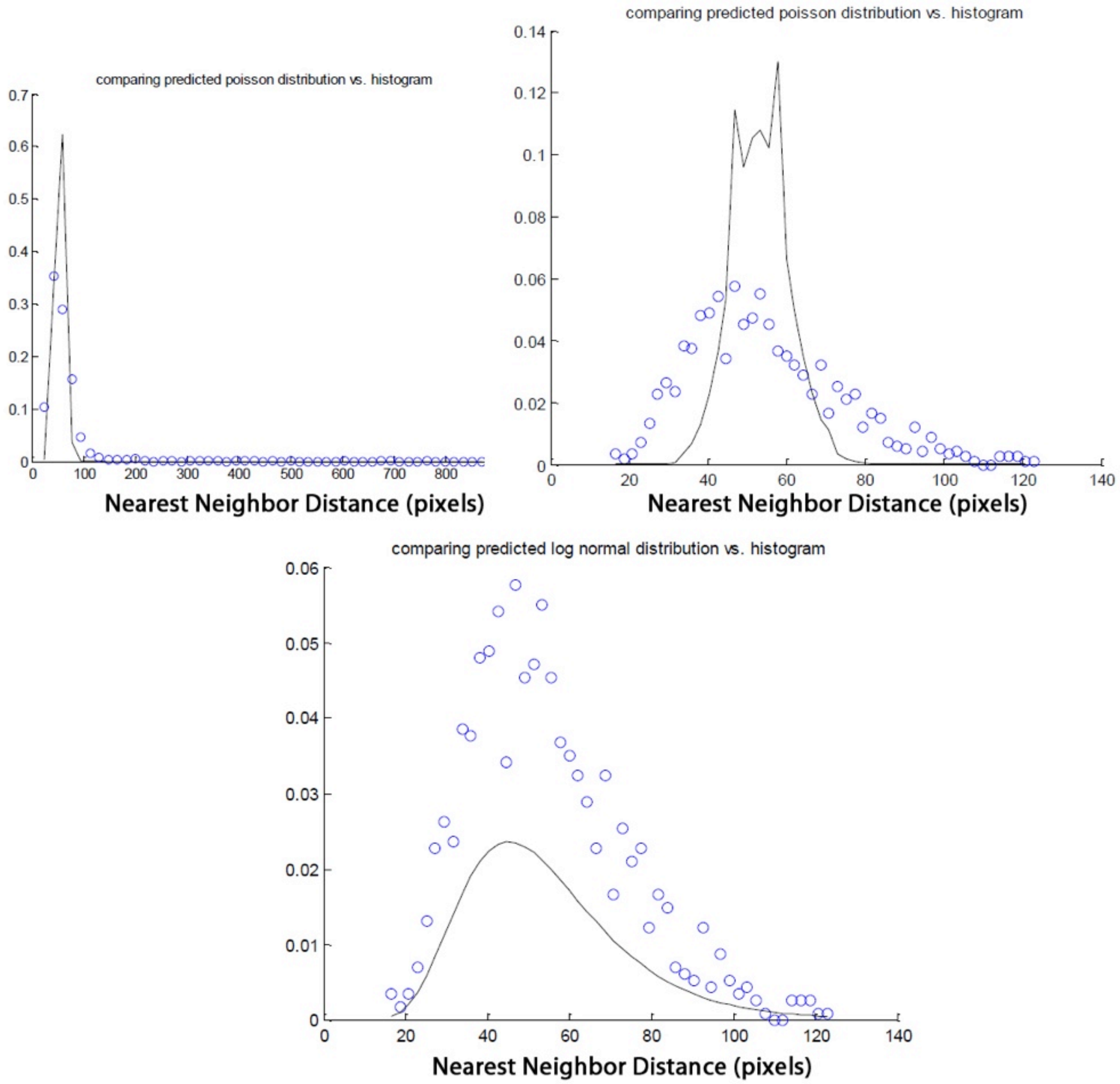


**Figure S10.** Sherini Ear: *Above left*: The histogram (black line) is fit to a Poisson distribution (blue circles). *Above right*: the new Poisson distribution with the imposed 125 distance cutoff. Note that in reality, the mean is to the right of what we would expect if elephant hair density was like a Poisson distribution. *Below*: The histogram (black line) is fit to a log normal distribution (blue circles). However, the observed elephant hair density variance in this case is too small. This is the opposite of the Poisson case where the observed variance was higher than the modeled variance.

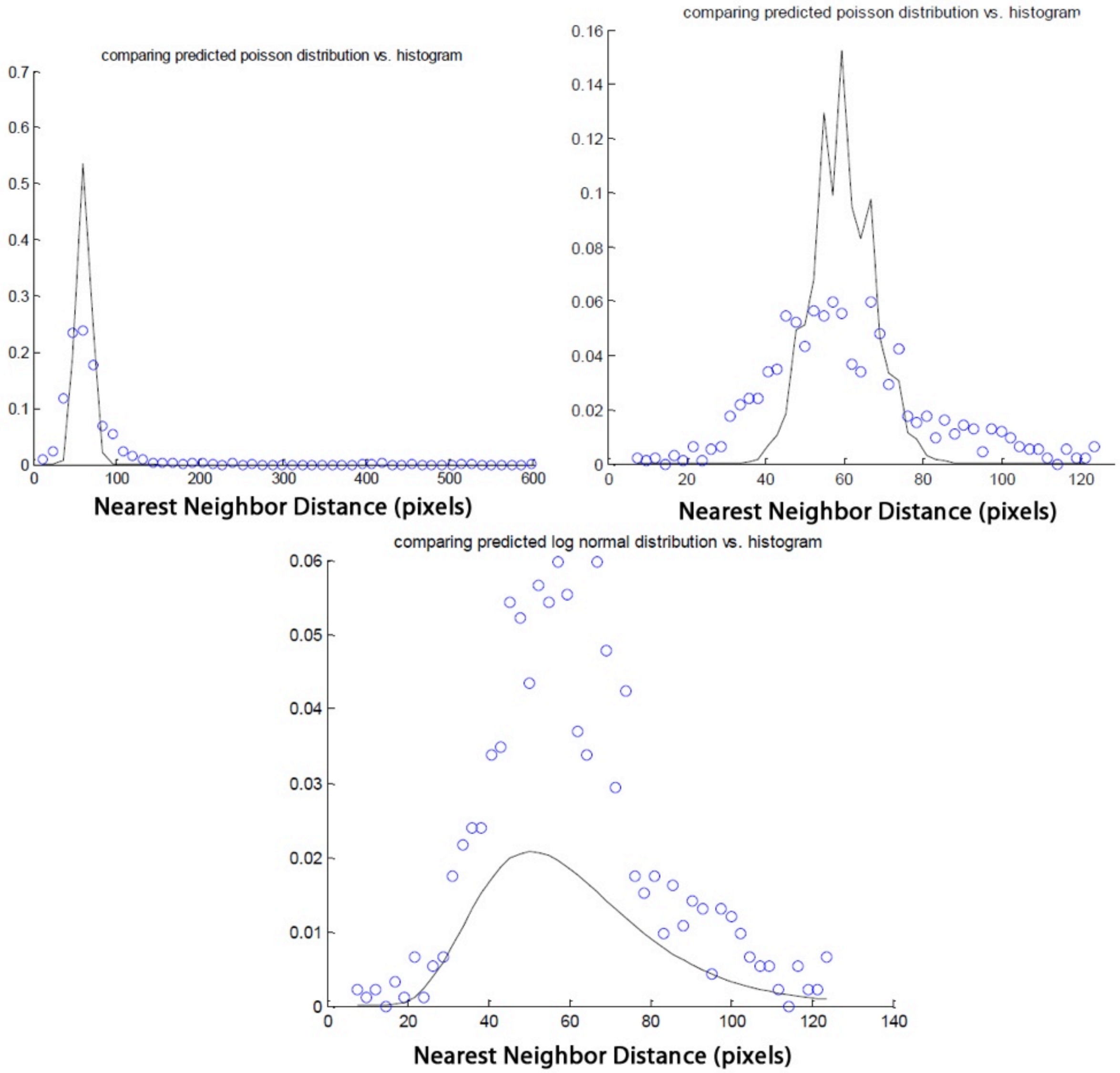


**Figure S11.** Sherini Leg: *Above left*: The histogram (black line) is fit to a Poisson distribution (blue circles). *Above right*: the new Poisson distribution with the imposed 125 distance cutoff. Note that in reality, the mean is to the right of what we would expect if elephant hair density was like a Poisson distribution. *Below*: The histogram (black line) is fit to a log normal distribution (blue circles). However, the observed elephant hair density variance in this case is too small. This is the opposite of the Poisson case where the observed variance was higher than the modeled variance.

## References for Supporting Information


1. Fowler A, Bejan A (1995) Forced convection from a surface covered with flexible fibers. International Journal of Heat and Mass Transfer 38: 767-777.
2. Lund KO (2001) Fully developed turbulent flow and heat transfer at fiber-flocked surfaces. International Journal of Heat and Mass Transfer 44: 3799-3810.
3. Lillywhite HB, Stein BR (1987) Surface sculpturing and water retention of elephant skin. Journal of Zoology, London 211: 727-734.
4. Phillips PK, Heath JE (1992) Heat exchange by the pinna of the African elephant (Loxodonta africana). Comparative Biochemistry and Physiology 101A: 693-699.
5. Valente A (1983) Hair structure of the Woolly mammoth, Mammuthus primigenius and the modern elephants, Elephas maximus and Loxodonta africana. Journal of Zoology, London 199: 271-274.
6. Weissenböck N, Arnold W, Ruf T Taking the heat: thermoregulation in Asian elephants under different climatic conditions. Journal of Comparative Physiology B: Biochemical, Systemic, and Environmental Physiology: 1-9.
7. Williams TM (1990) Heat transfer in elephants: thermal partitioning based on skin temperature profiles. Journal of Zoology, London 222: 235-245.
8. Pope SB (2000) Turbulent flows. Cambridge: Cambridge University Press. 771 p.
9. Brutsaert W (1982) Evaporation into the atmosphere: theory, history, and applications. Dordrecht, Holland: Reidel. 299 p.
10. Wright PJ (1984) Why do elephants flap their ears? South African Journal of Zoology 19: 267-269.
11. Weissenbock NM, Weiss CM, Schwammer HM, Kratochvil H (2010) Thermal windows on the body surface of African elephants (Loxodonta africana) studied by infrared thermography. Journal of Thermal Biology 35: 182-188.
12. Weissenbock NM, Arnold W, Ruf T (2012) Taking the heat: thermoregulation in Asian elephants under different climatic conditions. Journal of Comparative Physiology B-Biochemical Systemic and Environmental Physiology 182: 311-319.
13. Benedict FG, Lee RC (1938) Further Observations on the Physiology of the Elephant. Journal of Mammalogy 19: 175.
14. Tregear RT (1965) Hair Density Wind Speed and Heat Loss in Mammals. Journal of Applied Physiology 20: 796-&.
